# Supplementary material for: TRANSALUD: A qualitative study of the healthcare experiences of transgender people in Barcelona (Spain)
Source: PLoS One. 2022 Aug 3;17(8):e0271484. doi: 10.1371/journal.pone.0271484 (PMC9348718; doi:10.1371/journal.pone.0271484)
Supplement: Attachment — Submitted filename: Transalud Response to Reviewers 19062022.docx [file pone.0271484.s001.docx]

Dear editor,

I would like to submit a new version of the manuscript incorporating the reviewers’ suggestions which have undoubtedly helped to improve the manuscript. We have also included in the main text the two tables mentioned. A point-by-point response to each of the reviewers’ comments can be found below. I hope that this new version meets the requirements for publication in your journal.

Kind regards

| **Reviewer comment** | **Answer** | **Changes made** |
| --- | --- | --- |
| the introduction is quite long and sometimes goes out of the scope of the present analysis. I would suggest to summarise this paragraph. | Thank you for your suggestion.  The introduction has been shortened deleting some text that may be a bit out of the scope. |  |
| Why you hide the ethical committee if I have the list of authors in the first manuscript page? Please describe the ethical committee | The name of the IRB has been included in the text. | New sentence:  The study was approved by the ethical committee of the Universitat Autònoma de Barcelona (Spain). |
| Could you please introduce a reference for the following statement: “The data were analyzed descriptively and thematically following the method proposed by Colaizzi with the help of the Atlas.ti8 software.”? | We apologize for this missing reference. Thanks for your review. The reference has been added. | Colaizzi PF (1978): Psychological research as the phenomenologist views it. In: Valle RS, King M (eds) Existential phenomenological alternatives for psychology. Oxford University Press, New York. [[Google Scholar]](http://scholar.google.com/scholar?hl=en&q=Colaizzi+PF+%281978%29%3A+Psychological+research+as+the+phenomenologist+views+it.+In%3A+Valle+RS%2C+King+M+%28eds%29+Existential+phenomenological+alternatives+for+psychology.+Oxford+University+Press%2C+New+York.) |
| It would be useful to discuss the possible Health-care disparities stemming from sexual orientation according to disorders. Several papers have already described this topic and you could use them to discuss the possible different approach in different disciplines (see doi:10.1016/j.msard.2017.02.001; doi: 10.1016/j.cden.2021.06.007; doi: 10.1161/CIR.0000000000001003; doi: 10.1016/j.jaad.2018.02.042) | Thank you for your suggestion. Some new data has been added to the discussion regarding health disparities. | See the red text in the article. |
| The aim is quite ambitious, as the amount of manuscripts on the topic has steeply surged within the last year in relation with both healthy conditions and diseases. I would better highlight why authors decided to only include manuscript published between January and February 2021 and also I would further introduce the possible association between the physical exercise and quality of life, including the sleeping status, in both healthy controls and in patients with other diseases (see this works Durucan et al. 2022 [10.1097/MRR.0000000000000519]; Cunha et al. 2021 [10.1016/j.numecd.2021.12.019]; Carotenuto et al. 2021 [10.3390/jcm10061234]; Beydoun et al. 2022 [10.1093/gerona/glac028]; Bruno et al. 2022 [10.1016/j.sleep.2022.01.002]). All the aforementioned manuscript usually referred to people’s condition during the period you choose for selecting manuscript and hence, during the more severe COVID 19 restrictions. | We appreciate your comment. However, we believe there must be a mistake since we have included manuscripts from a wide publication year range. Moreover, we do not discuss in our paper anything about covid, exercise or other issues. All the references you suggest do not match with our research aim and findings. | - |
